# Stress, depression, anxiety, and quality of life among the healthcare workers during COVID-19 pandemic in Syria: a multi-center study

**DOI:** 10.1186/s12991-023-00470-1

**Published:** 2023-10-16

**Authors:** Hasan Nabil Al Houri, Abdullah Alhouri, Douaa Mohammad Nazir Arrouk, Ahmad Nabil Al Houri, Sami Jomaa, Alaa Sharabi, Hussein Kannout, Youssef Latifeh

**Affiliations:** 1 Internal Medicine Department, Damascus University, Damascus, Syria; 2 Division of Respiratory Medicine, Department of Medicine, Nevill Hall Hospital, Aneurin Bevan University Health Board, Wales, UK; 3 Applied Statistics – Quantitative Methods, Damascus University, Damascus, Syria; 4 Diagnostic Radiology Department, Damascus University, Damascus, Syria; 5 Faculty of Medicine, Damascus University, Damascus, Syria; 6 Somaerian Health, Abu Dhabi, UAE; 7 Center for Biotechnology, Khalifa University, Abu Dhabi, UAE; 8 Department of Psychiatry, Faculty of Medicine, Damascus University, Damascus, Syria

**Keywords:** Depression, Anxiety, Stress, Healthcare workers, Syria

## Abstract

**Background:**

The COVID-19 pandemic emerged as an expected source of stress and anxiety as the healthcare workers had to work for long hours in close contact with infected patients, thus increasing the probability of medical errors and threatening the patients’ safety. This study aims to measure the levels of depressive symptoms, anxiety symptoms, and stress among Syrian healthcare workers and their quality of life during the COVID-19 pandemic.

**Methods:**

A cross-sectional study was conducted in six central hospitals in Damascus, Syria. Data were collected from 1 to 30 June—2021. The Depression Anxiety Stress Scale—21 (DASS-21) was used to evaluate depression, anxiety, and stress among healthcare workers. Quality of life was assessed using the EUROHIS-QOL 8-item index.

**Results:**

A total of 700 participants were included in this study. 61.6% (*n* = 431) were males and 38.4% (*n* = 269) were females. Younger ages (18–29 years old) were significantly associated with higher levels of depression and stress (*p* < 0.0083). Female healthcare workers had higher significant levels of anxiety (*p* < 0.05). Significant anxiety and stress levels were reported when healthcare workers had contact with COVID-19 patients, even if they had protective equipment (*p* < 0.05). Half of the participants (50%; *n* = 349) reported a good quality of life.

**Conclusion:**

Stress levels and depressive symptoms were remarkably higher in healthcare workers of ages 18 and 29 years old, whereas anxiety levels were significantly higher and more severe in female healthcare workers. Moreover, direct interaction with COVID-19 patients was associated with higher levels of stress and anxiety symptoms.

## Introduction

Stress is a tense feeling caused by situations that jeopardize our stability. While anxiety is the fear and being uncomfortable of the anonymous. Alternatively, depression is a status of lack of interest and hopelessness [1]. Nonetheless, stress is a well-known factor in developing anxiety and depression [2]. We have learned from the Ebola and SARS epidemics that the abrupt onset of a severe and life-threatening infection could deteriorate the healthcare systems and render the physicians’ effectiveness [3]. The COVID-19 pandemic emerged as an inevitable source of stress and anxiety [4]. During this time, the healthcare workers had to work long shifts under enormous pressure to meet the demand of overcrowded wards and isolation rooms. While they were working in close contact with infected patients lacking personal protective equipment, they were prone to being infected, which in turn increased the probability of developing symptoms related to stress, anxiety, and depression [5–7]. Moreover, fears of transmitting the infection to their families and friends, lack of social support, and isolation and quarantine were major concerns for healthcare workers [8]. Even worse, healthcare workers may become more anxious and stressed when they develop signs and symptoms related to the infection [9].

It is obvious that psychological distress could increase medical errors and jeopardize the patients’ safety [10]; hence, the burden of COVID-19 goes beyond being “an emergent medical situation”. Thus, it is crucial to notice any dejection, irritability, self-blaming, or evading behaviors as early signs of mental distress [4] and to develop new strategies to cope with them [11], which would reduce the stress on healthcare workers and subsequently improve the patient's health [12, 13]. One Chinese study showed that 55.1%, 54.2%, and 58% of HCWs had symptoms of stress, anxiety, and depression, respectively, which emphasizes this situation of HCWs is worrying and intervention service is urgent [14]. Another study conducted in Egypt and Saudi Arabia reported that 69% of HCWs had depression, 58.9% had anxiety, and 55.9% had stress [15].

No research papers in Syria have been published that assess the psychological influence of COVID-19 on the public in general or health care professionals. Therefore, this study aims to measure the levels of depressive symptoms, anxiety symptoms, and stress among healthcare professionals, their quality of life during the COVID-19 pandemic, and the feasible methods to reduce stressful occurrences.

## Methods

### Study design and setting

The Syrian Ministry of Health designated Al Assad University Hospital, Al Mouwasat University Hospital, Children’s University Hospital, Maternal University Hospital, Dermatology University Hospital, and Al-Biruni University Hospital, the largest teaching hospitals in Syria, as dedicated centers for treating COVID-19 patients in Damascus. A cross-sectional study was conducted in six central hospitals in Damascus, Syria. Later, random samples were taken from the staff working in these hospitals, and they were contacted to complete the study questionnaire.

Data were collected from 1 to 30 June—2021. Healthcare workers were selected by survey method to enroll in a self-administered questionnaire. Male and female healthcare workers were eligible to participate in the study. Physicians, dentists, nurses, pharmacists, and other medical technicians were included.

The Depression Anxiety Stress Scale—21 (DASS-21) was used to evaluate depression, anxiety, and stress among healthcare workers [16]. The DASS-21 represents 21 self-reported items, divided into three scales containing seven items to assess depression, anxiety, and stress. The DASS-21 uses a four-point Likert scale fluctuating from 0 to 3: mild, moderate, severe, and extremely severe. Finally, quality of life was assessed using the EUROHIS-QOL 8-item index [17]. The respondents evaluated their quality of life as poor, very poor, neither poor nor good, good, and very good. The researchers sent the questionnaires to healthcare professionals either personally or via e-mail. A cover letter was attached to the questionnaire explaining the study, its goals, and how to complete and return the form. Participants were required to sign consent papers, and self-completed questionnaires were sent immediately to the researchers.

Participants provided their written informed consent, and anonymity and confidentiality were secured by providing each participant with a unique identification number that was only visible to the research team. The authors assert that all procedures contributing to this work comply with the ethical standards of the relevant national and institutional committees on human experimentation and with the Helsinki Declaration of 1975, as revised in 2008. All procedures involving human subjects/patients were approved by the institutional review board (IRB 613-2021).

### Statistical analysis

Statistical analysis was performed using SPSS Statistics version 23. Data were collected and exported into an Excel sheet. Qualitative and descriptive analysis was conducted to calculate frequencies, percentages, mean, and standard deviation for Quality of Life. Descriptive statistics were conducted to examine the mean and standard deviation of depression, anxiety, and stress scores. A cross-tabulation was performed between DASS and demographic variables. The chi-squared test of independence was used to study the association between qualitative variables. For our study, the Bonferroni adjustment method was employed as a correction strategy for multiple testing. Under the Bonferroni adjustment, a test’s significance is established only if its corresponding *p* value equals or less than α/*n*, where *n* represents the total number of distinct tests conducted on the same dataset. In our case, as there were six tests carried out for each dependent variable (depression, anxiety, and stress), the adjusted significance threshold was set at 0.05 divided by 6, yielding a value of 0.0083. As a result, each individual test's outcome was evaluated against this adjusted significance level of 0.0083, ensuring stringent control over the possibility of Type I errors arising from multiple comparisons.

## Results

A total of 700 participants were included in this study. 61.6% (*n* = 431) were males and 38.4% (*n* = 269) were females (Table 1). The age of 79.4% of the participants (*n* = 556) ranged from 18 to 29 years old. 71.4% (*n* = 500) were affiliated to the faculty of medicine. 59.5% (*n* = 417) of the participants had or were enrolling in a master's program; participants in internal medicine represented the majority (44.7%; *n* = 313) (Table 1). Descriptive statistics were conducted to examine the mean and standard deviation of depression, anxiety, and stress scores. The results revealed that individuals aged 50 and above had lower mean scores for depression (6.87), anxiety (5.07), and stress (11.60) (Table 2).

**Table 1:** Demographic characteristics

Variables		Frequency	Percentage
Age	18–29	556	79.4
	30–49	114	16.3
	> = 50	30	4.3
Gender	Male	269	38.4
	Female	431	61.6
Place of residence	Damascus	474	67.7
	Rif Dimashq	123	17.6
	Other	103	14.7
Educational level	Doctorate	23	3.3
	Master degree	417	59.6
	Diploma	112	16
	University degree	148	21.1
Field of studying	Medicine	500	71.4
	Dentistry	16	2.3
	Pharmacy	40	5.7
	Nursing	130	18.6
	Medical institute	14	2
Medical specialty	General surgery	38	5.4
	Internal medicine	313	44.7
	Obstetrics and gynecology	24	3.4
	Paediatric	14	2
	Special medicine	58	8.3
	Special surgery	53	7.6
	Dentistry	16	2.3
	Nursing	144	20.6
	Pharmacist	38	5.4
	Pharmacy	2	0.3

*N* = 700

**Table 2:** Depression, anxiety and stress mean and standard deviation among studies variable

Variable	Categories	Depression		Anxiety		Stress	
		Mean	Std. deviation	Mean	Std. deviation	Mean	Std. deviation
Age	18–29	16.05	10.772	10.04	8.240	18.30	10.593
	30–49	10.70	10.613	7.28	8.223	14.18	10.321
	> = 50	6.87	6.004	5.07	6.721	11.60	10.040
Gender	Male	14.25	11.001	8.12	8.138	16.24	10.434
	Female	15.12	10.813	10.17	8.282	18.03	10.802
Place of residence	Other	14.37	11.187	9.17	8.404	16.93	10.947
	Damascus	14.66	10.798	9.20	8.134	17.40	10.584
	Rif Dimashq	15.59	11.020	10.26	8.740	17.46	10.961
Financial status	Low	19.28	10.712	12.00	7.415	20.22	10.586
	Medium	14.64	11.113	8.83	8.121	16.55	10.862
	Good	14.32	10.608	9.45	8.546	17.80	10.478
	Excellent	10.63	9.523	7.38	7.712	12.88	9.902
Educational level	Doctorate	8.96	9.063	5.91	7.988	11.74	10.640
	Master degree	16.16	11.150	9.30	8.247	18.46	10.733
	Diploma	9.95	8.853	8.02	6.920	12.36	9.362
	University degree	15.47	10.548	11.18	9.020	18.84	10.230
Field of studying	Medicine	15.79	10.959	9.30	8.283	18.44	10.591
	Dentistry	10.75	10.655	9.00	8.548	13.38	10.112
	Pharmacy	16.35	12.477	11.35	9.548	17.70	11.578
	Nursing	11.20	9.326	9.06	7.785	13.71	9.918
	Medical institute	12.14	9.906	10.00	8.979	15.14	11.812
Medical specialty	General surgery	14.05	12.130	7.63	8.857	14.84	11.137
	Internal medicine	16.22	10.918	9.79	8.383	19.11	10.843
	Obstetrics and gynecology	18.00	11.451	11.33	7.993	20.58	9.353
	Paediatric	17.00	9.977	10.00	9.013	21.00	9.695
	Special medicine	15.00	11.102	9.10	8.412	17.52	10.743
	Special surgery	14.04	10.239	6.72	6.517	16.45	8.536
	Dentistry	10.75	10.655	9.00	8.548	13.38	10.112
	Nursing	11.29	9.352	9.15	7.879	13.85	10.080
	Pharmacist	16.84	12.508	11.89	9.483	18.26	11.495
	Pharmacy	7.00	9.899	1.00	1.414	7.00	9.899
How often did you have to work at the hospital or clinic (including private practice)?	Never	13.56	11.008	10.00	8.837	16.29	11.305
	Once a week	13.58	10.221	8.79	8.817	17.74	11.561
	2–3 days a week	14.87	10.682	9.06	8.072	16.87	10.530
	4–5 days a week	14.85	11.045	8.82	7.730	17.40	10.452
	All week days including weekend	15.55	11.108	11.00	9.185	18.50	10.918
If you worked at the hospital or clinic did other people such as neighbours, relatives, or co-workers know that you did?	No	17.38	10.693	12.56	8.065	20.92	9.379
	Yes	14.65	10.948	9.17	8.266	17.10	10.764
How extensive was your content with people infected with COVID-19?	No contact at all	9.64	8.958	5.64	6.891	12.45	10.918
	Only occasional contact for a few minutes with protective equipment	14.25	11.047	8.65	8.214	16.40	10.694
	Close daily contact but with protective equipment	15.85	10.704	10.59	8.295	18.88	10.484
When vaccine is available	I won’t take it	15.78	11.329	10.50	9.311	18.70	10.931
	I’ll take it but I’m worry	15.10	10.996	9.58	8.130	17.69	10.877
	I’ll take it and I’m reassured	13.72	10.616	8.38	7.722	16.07	10.341

**Fig. 1:**
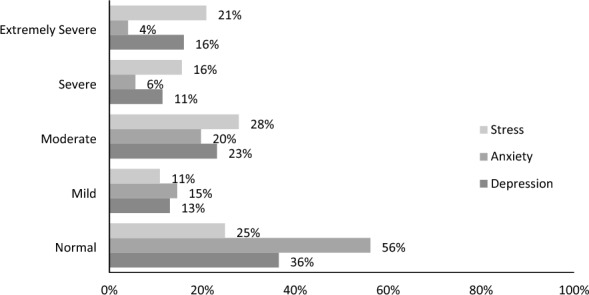
Illustration of the frequency of depression, anxiety and stress among 700 participants and the association of different levels of them

Anxiety levels (Fig 1) illustrates the distribution of responses: 36.4% (*n* = 255) displayed no depression signs or symptoms, while 36.1% (*n* = 253) showed mild to moderate levels. Severe depression was indicated by 11.4% (*n* = 80), and 16.0% (*n* = 112) exhibited extremely severe depression. For anxiety, 56.1% (*n* = 393) had no symptoms, 34.3% (*n* = 240) displayed mild to moderate levels, 5.6% (*n* = 39) had severe symptoms, and 4.0% (*n* = 28) were extremely severe. In terms of stress, 24.9% (*n* = 174) showed no signs, while 15.6% (*n* = 109) had severe and 2.9% (*n* = 146) had extremely severe stress levels.

A comparison between variables using chi-squared was performed to calculate the significant level of the variables. Younger ages (18–29 years old) were significantly associated with higher levels of depression and stress (*p* < 0.0083). Regarding depression, 12.8% (*n* = 71) of participants in this age group had severe levels, while 18.0% (*n* = 100) had extremely severe levels (Table 3).

**Table 3:** Meta-analyzed results of the participants’ demographics and their association with depression

Variables			Depression					
			Normal	Mild	Moderate	Severe	Extremely severe	Chi-squared test (*p* value)
Age	18–29	*n*	168	75	142	71	100	**0.000**
		%	30.20%	13.50%	25.50%	12.80%	18.00%	
	30–49	*n*	68	8	18	8	12	
		%	59.60%	7.00%	15.80%	7.00%	10.50%	
	≥ 50	*n*	19	8	2	1	0	
		%	63.30%	26.70%	6.70%	3.30%	0.00%	
	Total	*n*	255	91	162	80	112	
		%	36.40%	13.00%	23.10%	11.40%	16.00%	
Gender	Males	*n*	74	33	77	40	45	0.212
		%	27.50%	12.30%	28.60%	14.90%	16.70%	
	Females	*n*	100	43	118	69	101	
		%	23.20%	10.00%	27.40%	16.00%	23.40%	
	Total	*n*	174	76	195	109	146	
		%	24.90%	10.90%	27.90%	15.60%	20.90%	
Educational level	Doctorate	*n*	13	3	4	2	1	**0.0019**
		%	56.50%	13.00%	17.40%	8.70%	4.30%	
	Master degree	*n*	135	53	99	48	82	
		%	32.40%	12.70%	23.70%	11.50%	19.70%	
	Diploma	*n*	59	15	22	12	4	
		%	52.70%	13.40%	19.60%	10.70%	3.60%	
	University degree	*n*	48	20	37	18	25	
		%	32.40%	13.50%	25.00%	12.20%	16.90%	
	Total	*n*	255	91	162	80	112	
		%	36.40%	13.00%	23.10%	11.40%	16.00%	
Field of studying	Medicine	*n*	167	65	116	60	92	0.015
		%	33.40%	13.00%	23.20%	12.00%	18.40%	
	Dentistry	*n*	8	1	5	1	1	
		%	50.00%	6.30%	31.30%	6.30%	6.30%	
	Pharmacy	*n*	13	4	10	2	11	
		%	32.50%	10.00%	25.00%	5.00%	27.50%	
	Nursing	*n*	59	21	29	14	7	
		%	45.40%	16.20%	22.30%	10.80%	5.40%	
	Medical institute	*n*	8	0	2	3	1	
		%	57.10%	0.00%	14.30%	21.40%	7.10%	
	Total	*n*	255	91	162	80	112	
		%	36.40%	13.00%	23.10%	11.40%	16.00%	
If you worked at the hospital or clinic did other people such as neighbours, relatives, or co-workers know that you did?	No	*n*	11	5	7	6	10	0.413
		%	28.20%	12.80%	17.90%	15.40%	25.60%	
	Yes	*n*	242	85	148	74	102	
		%	37.20%	13.10%	22.70%	11.40%	15.70%	
	Total	*n*	253	90	155	80	112	
		%	36.70%	13.00%	22.50%	11.60%	16.20%	
How extensive was your content with people infected with COVID-19?	No contact at all	*n*	12	3	4	2	1	0.308
		%	54.50%	13.60%	18.20%	9.10%	4.50%	
	Occasional contact for a few minutes with protective equipment	*n*	146	48	79	44	56	
		%	39.10%	12.90%	21.20%	11.80%	15.00%	
	Close daily contact but with protective equipment	*n*	96	40	78	34	55	
		%	31.70%	13.20%	25.70%	11.20%	18.20%	
	Total	*n*	254	91	161	80	112	
		%	36.40%	13.00%	23.10%	11.50%	16.00%	

The *p* value were significant at < 0.0083 and were highlighted in bold

**Table 4:** Meta-analyzed results of the participants’ demographics and its association with anxiety

Variables			Anxiety					
			Normal	Mild	Moderate	Severe	Extremely severe	Chi-squared test (p value)
Age	18–29	*n*	293	89	116	34	24	0.051
		%	52.70%	16.00%	20.90%	6.10%	4.30%	
	30–49	*n*	76	11	19	4	4	
		%	66.70%	9.60%	16.70%	3.50%	3.50%	
	≥ 50	*n*	24	2	3	1	0	
		%	80.00%	6.70%	10.00%	3.30%	0.00%	
	Total	*n*	393	102	138	39	28	
		%	56.10%	14.60%	19.70%	5.60%	4.00%	
Gender	Males	*n*	174	32	39	14	10	**0.007**
		%	64.70%	11.90%	14.50%	5.20%	3.70%	
	Females	*n*	219	70	99	25	18	
		%	50.80%	16.20%	23.00%	5.80%	4.20%	
	Total	*n*	393	102	138	39	28	
		%	56.10%	14.60%	19.70%	5.60%	4.00%	
Educational level	Doctorate	*n*	16	2	4	1	0	0.07
		%	69.60%	8.70%	17.40%	4.30%	0.00%	
	Master degree	*n*	245	52	79	24	17	
		%	58.80%	12.50%	18.90%	5.80%	4.10%	
	Diploma	*n*	65	22	17	7	1	
		%	58.00%	19.60%	15.20%	6.30%	0.90%	
	University degree	*n*	67	26	38	7	10	
		%	45.30%	17.60%	25.70%	4.70%	6.80%	
	Total	*n*	393	102	138	39	28	
		%	56.10%	14.60%	19.70%	5.60%	4.00%	
Field of studying	Medicine	*n*	291	63	100	26	20	0.163
		%	58.20%	12.60%	20.00%	5.20%	4.00%	
	Dentistry	*n*	9	4	1	1	1	
		%	56.30%	25.00%	6.30%	6.30%	6.30%	
	Pharmacy	*n*	18	4	12	3	3	
		%	45.00%	10.00%	30.00%	7.50%	7.50%	
	Nursing	*n*	68	30	21	7	4	
		%	52.30%	23.10%	16.20%	5.40%	3.10%	
	Medical Institute	*n*	7	1	4	2	0	
		%	50.00%	7.10%	28.60%	14.30%	0.00%	
	Total	*n*	393	102	138	39	28	
		%	56.10%	14.60%	19.70%	5.60%	4.00%	
If you worked at the hospital or clinic did other people such as neighbours, relatives, or co-workers know that you did?	No	*n*	14	5	13	6	1	**0.008**
		%	35.90%	12.80%	33.30%	15.40%	2.60%	
	Yes	*n*	375	95	122	32	27	
		%	57.60%	14.60%	18.70%	4.90%	4.10%	
	Total	*n*	389	100	135	38	28	
		%	56.40%	14.50%	19.60%	5.50%	4.10%	
How extensive was your content with people infected with COVID-19?	No contact at all	*n*	15	1	6	0	0	0.011
		%	68.20%	4.50%	27.30%	0.00%	0.00%	
	Occasional contact for a few minutes with protective equipment	*n*	227	56	55	18	17	
		%	60.90%	15.00%	14.70%	4.80%	4.60%	
	Close daily contact but with protective equipment	*n*	149	45	77	21	11	
		%	49.20%	14.90%	25.40%	6.90%	3.60%	
	Total	*n*	391	102	138	39	28	
		%	56.00%	14.60%	19.80%	5.60%	4.00%	

The *p* value were significant at < 0.0083 and were highlighted in bold

Medium/good financial status is significantly associated with severe/extremely severe levels of depression and anxiety (p < 0.0083) (Tables 3 and 4). Educational level was significantly associated with depression and stress (*p* < 0.0083). 68.8% (*n* = 287) of Master students had normal to moderate levels of depression, 11.5% (*n* = 48) had severe levels, and 19.7% (*n* = 82) had extremely severe levels of depression (Table 3). Furthermore, 58.8% of Master students had normal to moderate levels of stress, 18% (*n* = 75) had severe levels, and 23.3% (*n* = 97) had extremely severe levels of stress (Table 5). Field of study was also significantly associated with stress (*p* < 0.0083). 69.6% (*n* = 348) of participants who studied medicine had normal to moderate levels of depression, 12.0% (*n* = 60) had severe levels, and 18.4% (*n* = 92) had extremely severe levels of depression (Table 3). In addition, 58.2% (*n* = 294) of those who studied medicine had normal to moderate levels of stress, 17.6% (*n* = 88) had severe levels, and 23.6% (*n* = 118) had extremely severe levels of stress (Table 5).

**Table 5:** Meta-analyzed results of the participants’ demographics and its association with stress

Variables			Stress					
			Normal	Mild	Moderate	Severe	Extremely severe	Chi-squared test (p value)
Age	18–29	*n*	118	61	155	97	125	**0.001**
		%	21.20%	11.00%	27.90%	17.40%	22.50%	
	30–49	*n*	42	11	32	12	17	
		%	36.80%	9.60%	28.10%	10.50%	14.90%	
	≥ 50	*n*	14	4	8	0	4	
		%	46.70%	13.30%	26.70%	0.00%	13.30%	
	Total	*n*	174	76	195	109	146	
		%	24.90%	10.90%	27.90%	15.60%	20.90%	
Gender	Males	*n*	106	39	52	28	44	0.288
		%	39.40%	14.50%	19.30%	10.40%	16.40%	
	Females	*n*	149	52	110	52	68	
		%	34.60%	12.10%	25.50%	12.10%	15.80%	
	Total	*n*	255	91	162	80	112	
		%	36.40%	13.00%	23.10%	11.40%	16.00%	
Educational level	Doctorate	*n*	11	3	4	3	2	**0.000**
		%	47.80%	13.00%	17.40%	13.00%	8.70%	
	Master degree	*n*	88	45	112	75	97	
		%	21.10%	10.80%	26.90%	18.00%	23.30%	
	Diploma	*n*	48	14	33	8	9	
		%	42.90%	12.50%	29.50%	7.10%	8.00%	
	University degree	*n*	27	14	46	23	38	
		%	18.20%	9.50%	31.10%	15.50%	25.70%	
	Total	*n*	174	76	195	109	146	
		%	24.90%	10.90%	27.90%	15.60%	20.90%	
Field of studying	Medicine	*n*	103	55	136	88	118	**0.002**
		%	20.60%	11.00%	27.20%	17.60%	23.60%	
	Dentistry	*n*	6	2	4	3	1	
		%	37.50%	12.50%	25.00%	18.80%	6.30%	
	Pharmacy	*n*	9	4	13	5	9	
		%	22.50%	10.00%	32.50%	12.50%	22.50%	
	Nursing	*n*	49	15	41	11	14	
		%	37.70%	11.50%	31.50%	8.50%	10.80%	
	Medical Institute	*n*	7	0	1	2	4	
		%	50.00%	0.00%	7.10%	14.30%	28.60%	
	Total	*n*	174	76	195	109	146	
		%	24.90%	10.90%	27.90%	15.60%	20.90%	
If you worked at the hospital or clinic did other people such as neighbours, relatives, or co-workers know that you did?	No	*n*	4	4	11	9	11	0.186
		%	10.30%	10.30%	28.20%	23.10%	28.20%	
	Yes	*n*	169	70	181	100	131	
		%	26.00%	10.80%	27.80%	15.40%	20.10%	
	Total	*n*	173	74	192	109	142	
		%	25.10%	10.70%	27.80%	15.80%	20.60%	
How extensive was your content with people infected with COVID-19?	No contact at all	*n*	12	0	4	4	2	**0.006**
		%	54.50%	0.00%	18.20%	18.20%	9.10%	
	Occasional contact for a few minutes with protective equipment	*n*	103	44	99	58	69	
		%	27.60%	11.80%	26.50%	15.50%	18.50%	
	Close daily contact but with protective equipment	*n*	59	31	91	47	75	
		%	19.50%	10.20%	30.00%	15.50%	24.80%	
	Total	*n*	174	75	194	109	146	
		%	24.90%	10.70%	27.80%	15.60%	20.90%	

The *p* value were significant at < 0.0083 and were highlighted in bold

Specialty of physicians was also associated with significant levels of stress. 56.9% (*n* = 178) of internal medicine physicians had normal to moderate levels of depression, 16.9% (*n* = 53) had severe levels, and 26.2% (*n* = 82) had extremely severe levels of stress (Table 5). Healthcare workers reported significant anxiety levels when their neighbours, relatives, or co-workers knew that they had worked in the hospital (*p* < 0.0083). They also reported significant levels of stress when they had a contact with COVID-19 patients (*p* < 0.0083).

**Fig. 2:**
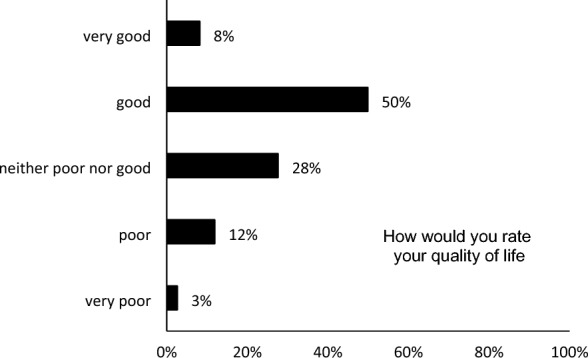
The quality of life among healthcare workers

Finally, quality of life was assessed through eight questions. Half of the participants (50%; *n* = 349) reported a good QoL. 28% (*n* = 193) reported that their QoL was neither good nor bad. 12% (*n* = 38) had poor QoL, and 8% (*n* = 57) had a very good QoL (Fig. 2, Table 6).

**Table 6:** DASS ranges score

DASS ranges score			
	Depression	Anxiety	Stress
Normal	0–9	0–7	0–14
Mild	10 _ 13	8_9	15–18
Moderate	14–20	10_14	19–25
Severe	21–27	15–19	26–33
Extremely severe	28 +	20 +	34 +

## Discussion

The impact of depression, anxiety, and stress on healthcare workers (HCWs) performance is widely acknowledged, posing a risk to patient well-being. With the emergence of the COVID-19 pandemic, it has become crucial to assess the influence of this crisis on the mental health of Syrian HCWs.

Our study revealed that female HCWs had significantly higher and more severe levels of anxiety (*p* < 0.0083) when compared to males HCWs, 5.8% vs. 5.2% had severe anxiety levels and 4.2% vs. 3.7% had extremely severe levels, respectively. Although our study found that gender have no impact on depression and stress levels, Lai et al. [16, 18] and Rossi et al. [17, 19] reported that women had more severe symptoms in three aspects of DASS-21. Contrary, Suryavanshi et al. [18, 20] found no association between gender and risk of mental distress.

We noticed that healthcare workers aged 18–29 (*n* = 556/700) had significantly elevated levels of depression and stress (*p* < 0.0083), although their anxiety levels were not significantly different. A study conducted in Spain yielded similar results, showing that individuals aged 18–25 (*n* = 551/976) experienced higher levels of depression, anxiety, and stress. Many healthcare workers in this age group were also university students who had to transition from in-person learning to online platforms like Zoom and Google Meet, which may have contributed to their mental health challenges [11].

During the pandemic, the healthcare workers worked in close contact with infected patients for extensive hours and under an increased volume of pressure. They were susceptible to infection, leading to further mental strains on HCWs [5–7, 21, 22]. HCWs who were in direct contact with COVID-19 patients had depression and anxiety prevalence of 47 and 50%, respectively [20]. Our study showed that caring for COVID-19 patients would increase stress levels among HCWs, regardless of whether they had or did not have protective equipment. Lenzo et al. [23] and Rossi et al. [19] documented higher rates of moderate, severe, and extremely severe levels of depression, anxiety, and stress among HCWs who worked with COVID-19 patients. Lai et al. [18] reported that the first-line HCWs responsible for COVID-19 patients had an elevated risk of developing mental distress compared to those in the second-line. A study compared the prevalence of mental distress between a hospital that admits COVID-19 patients and those that do not. As expected, HCWs in the COVID-19-admitting hospital had higher rates of depression, anxiety, and stress [24]. It is worth mentioning that Hummel et al. [10] found no significant association between direct contact with COVID-19 patients and anxiety, depression, or stress levels among medical professionals.

Our study revealed that healthcare workers, like many others, have been affected by the pandemic in terms of their quality of life. Out of the participants, 50% (*n* = 349) reported having a good quality of life. Additionally, 28% (*n* = 193) stated that their quality of life was neither good nor bad, while 12% (*n* = 38) reported having a poor quality of life, and 8% (*n* = 57) reported having a very good quality of life. ).

Suryavanshi et al. [20] assessed QoL using one-item quality of life (QoL-1) visual analogue scale. They conveyed that moderate to severe depression and anxiety were independently associated with low QoL. A study from Vietnam reported a low health-related QoL among HCWs who had direct contact with COVID-19 patients [24]. Another Vietnamese study anticipated a low health-related QoL in people suspected of COVID-19 (25). Finally, it is essential to highlight the impact of the pandemic on the general population. Hummel et al. conducted a study across eight European countries, examining the mental health of both medical and non-medical professionals. Their findings revealed that healthcare workers had lower rates of depression and anxiety compared to non-medical professionals. It is crucial for researchers to shed light on the pandemic and its effects on the shed light impact suggested that their medical knowledge may helped them to understand the pandemic and be able to cope with it [10].

To summarize, this study has certain limitations as it only suggests associations rather than definitive cause-and-effect relationships. There is a possibility of reporting bias as the data relied on self-reported information from healthcare workers, whom the challenging circumstances of the pandemic may have influenced. Furthermore, the study solely focused on healthcare workers and did not consider the mental well-being of the general population. Therefore, future longitudinal studies should be conducted to explore the levels and underlying causes of depression, anxiety, stress, and quality of life among healthcare workers and compare them to those of the general population.20:07.

## Conclusion

Levels of stress and depressive symptoms were remarkably higher in HCWs between 18 and 29 years old, whereas anxiety symptoms levels were significantly higher and more severe in female HCWs. Extensive contact with COVID-19 patients was associated with higher stress levels.

## Data Availability

The datasets used and/or analyzed during the current study are available from the corresponding author upon reasonable request.
